# Supplementary material for: Effect of Switching to Once-Weekly Semaglutide on Non-Alcoholic Fatty Liver Disease: The SWITCH-SEMA 1 Subanalysis
Source: Pharmaceutics. 2023 Aug 20;15(8):2163. doi: 10.3390/pharmaceutics15082163 (PMC10459529; doi:10.3390/pharmaceutics15082163)
Supplement: Supplementary file 1 [file pharmaceutics-15-02163-s001.zip › Supple Tables_20230817.pdf]

Table S1. Relationship between the percent changes in the fatty liver index and changes in metabolic parameters

	**Continue (n=27)**		**Switch to semaglutide (n=31)**	
	**Correlation coefficient**	***P*-value**	**Correlation coefficient**	***P*-value**
ΔFPG	0.479	0.012	0.123	0.509
ΔHbA1c	0.418	0.030	0.061	0.745
ΔBMI	0.355	0.070	0.387	0.031
ΔAST	0.351	0.073	0.096	0.606
ΔALT	0.353	0.071	0.046	0.807
ΔeGFR	−0.024	0.906	−0.247	0.180
ΔTotal cholesterol	0.253	0.202	0.281	0.126

ALT, alanine aminotransferase; AST, aspartate aminotransferase; BMI, body mass index; eGFR, estimated glomerular filtration rate; FPG, fasting plasma glucose; HbA1c, glycated hemoglobin.

Table S2. Comparison of changes in representative metabolic parameters between the treatment arms

		Liraglutide (n=23)		Dulaglutide (n=35)		
		**Continue (n=10)**	**SWITCH (n=13)**	**Continue (n=17)**	**SWITCH (n=18)**	***P*-value**
**FLI**	**Baseline**	73.9 ± 22.5	71.2 ± 22.9	69.5 ± 16.2	66.6 ± 26.0	
	**Changes**	−1.41 (−7.04, 4.22)	−2.09 (−8.75, 4.57)	2.69 (−2.05, 7.44)	−8.64 (−12.28, −5.00) ***	0.056
**HbA1c (%)**	**Baseline**	7.6 ± 0.7	8.0 ± 0.8	7.9 ± 1.0	7.9 ± 0.7	
	**Changes**	0.0 (−0.3, 0.3)	−0.8 (−1.0, −0.6) ***	0.2 (−0.1, 0.5)	−0.9 (−1.2, −0.5) ***	0.699
**BMI (kg/m^2)**	**Baseline**	32.2 ± 6.1	32.1 ± 6.2	30.0 ± 2.6	30.5 ± 4.9	
	**Changes**	−0.1 (−0.5, 0.3)	−0.8 (−1.4, −0.2) *	0.2 (−0.2, 0.5)	−1.0 (−1.3, −0.7) ***	0.491

Values are presented as mean ± SD or mean (95% confidence interval). The significance of differences between 0 wks and 24 wks was assessed using Student's *t*-test. The significance of differences between continue and switch groups was assessed using unpaired *t*-test. *** $P < 0.001$ *vs* baseline. *P*-value: The significance of differences between switch from liraglutide and switch from dulaglutide, unpaired *t*-test.

Table S3. Relationships between changes in the fatty liver index and clinical parameters in the SWITCH group according to multiple linear regression analysis

	**Regression coefficients**	**95% confidence interval**	***P*-value**
**Age**	−0.10	−0.16, 0.19	0.569
**Gender (female = 1)**	2.28	−1.57, 6.1	0.234
**Fatty liver index**	0.02	−0.16, 0.19	0.855
**Switch from dulaglutide**	3.54	0.16, 6.91	0.041

Multiple linear regression was adjusted for age, gender (female = 1), baseline fatty liver index, and switch from dulaglutide.

Table S4. Comparison of baseline characteristics between switching from liraglutide and switching from dulaglutide in the SWITCH group

Variables	Switch from Lira to Sema (n=13)	Switch from Dula to Sema (n=18)	*P*-value
**Age (years old)**	61.2 ± 12.4	61.7 ± 13.4	0.989
**Female sex, n (%)**	6 (46.2)	6 (33.3)	0.710
**Body mass index (kg/m^2)**	32.1 ± 6.2	30.5 ± 4.9	0.423
**Waist circumference (cm)**	109.8 ± 10.3	105.4 ± 11.6	0.292
**HbA1c (%)**	8.0 ± 0.8	7.9 ± 0.8	0.876
**Triglyceride (mg/dL)**	111 (83-172)	119 (80-165)	0.508
**AST (IU/L)**	21 (21-28)	22 (18-35)	0.729
**ALT (IU/L)**	27 (19-33)	29 (21-39)	0.367
**γ-GTP (IU/L)**	21 (16-49)	31 (20-46)	0.458
**eGFR (mL/min/1.73m^2)**	67.4 ± 20.4	75.1 ± 24.1	0.358
**Fatty liver index**	71.2 ± 22.9	66.6 ± 26.0	0.614

Values are presented as mean ± SD, median (25-75 percentile), or number (%). *P*-value: The significance of differences between the groups, unpaired *t*-test, Mann-Whitney *U*-test, or Fisher's exact test. γ-GTP, γ-glutamyl transpeptidase; ALT, alanine aminotransferase; AST, aspartate aminotransferase; Dula, dulaglutide; eGFR, estimated glomerular filtration rate; HbA1c, glycated hemoglobin; Lira, liraglutide; Sema, semaglutide.

Table S5. MANOVA repeated measures results for fatty liver index.

Factors	F value	*P*-value
**Between subject**		
Treatment strategies (Continue GLP-RAs or switch to sema) (T)	3.27	0.077
Baseline GLP-1RA regimens (lira or dula) (B)	0.27	0.608
Age	1.56	0.218
BMI	39.83	< 0.001
sBP	2.38	0.130
HbA1c	4.96	0.031
T*B	0.47	0.495
T*Age	1.81	0.185
T*BMI	0.03	0.860
T*sBP	1.15	0.289
T*HbA1c	4.20	0.046
**Within subject**		
Period*T	4.15	0.048
Period*B	0.21	0.651
Period*Age	1.89	0.176
Period*BMI	0.00	1.000
Period*sBP	1.25	0.269
Period*HbA1c	0.14	0.714
Period*T*B	3.16	0.082
Period*T*Age	0.02	0.901
Period*T*BMI	0.28	0.602
Period*T*sBP	0.25	0.616
Period*T*HbA1c	0.28	0.601

BMI, body mass index; dula, dulaglutide; GLP-1RA, glucagon like peptide receptor agonist; HbA1c, glycated hemoglobin; lira, liraglutide; sema, semaglutide.
