# Supplementary figures and images for: Effect of Switching to Once-Weekly Semaglutide on Non-Alcoholic Fatty Liver Disease: The SWITCH-SEMA 1 Subanalysis
Source: Pharmaceutics. 2023 Aug 20;15(8):2163. doi: 10.3390/pharmaceutics15082163 (PMC10459529; doi:10.3390/pharmaceutics15082163)

## Slide 1
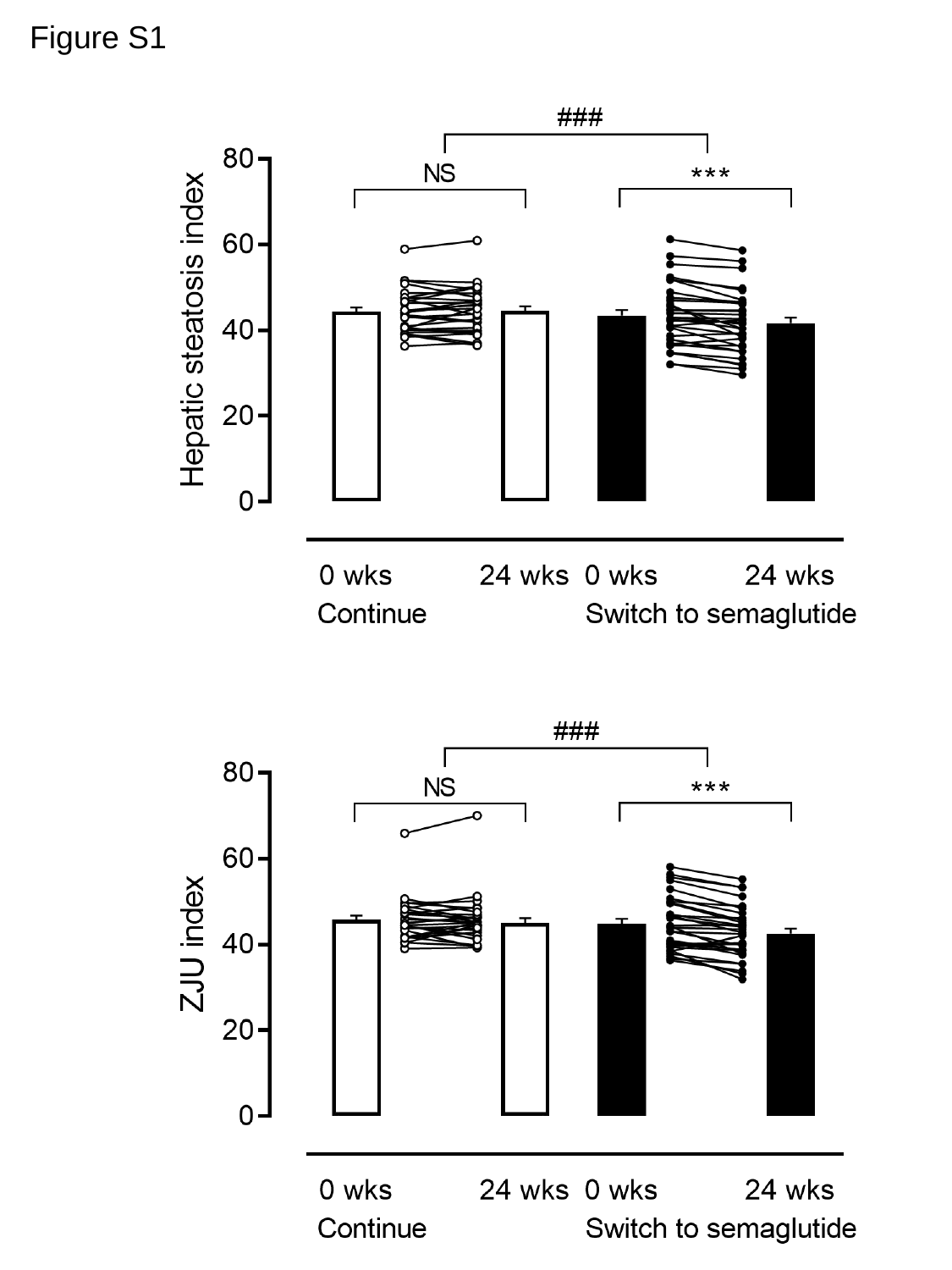

Figure S1

## Slide 2
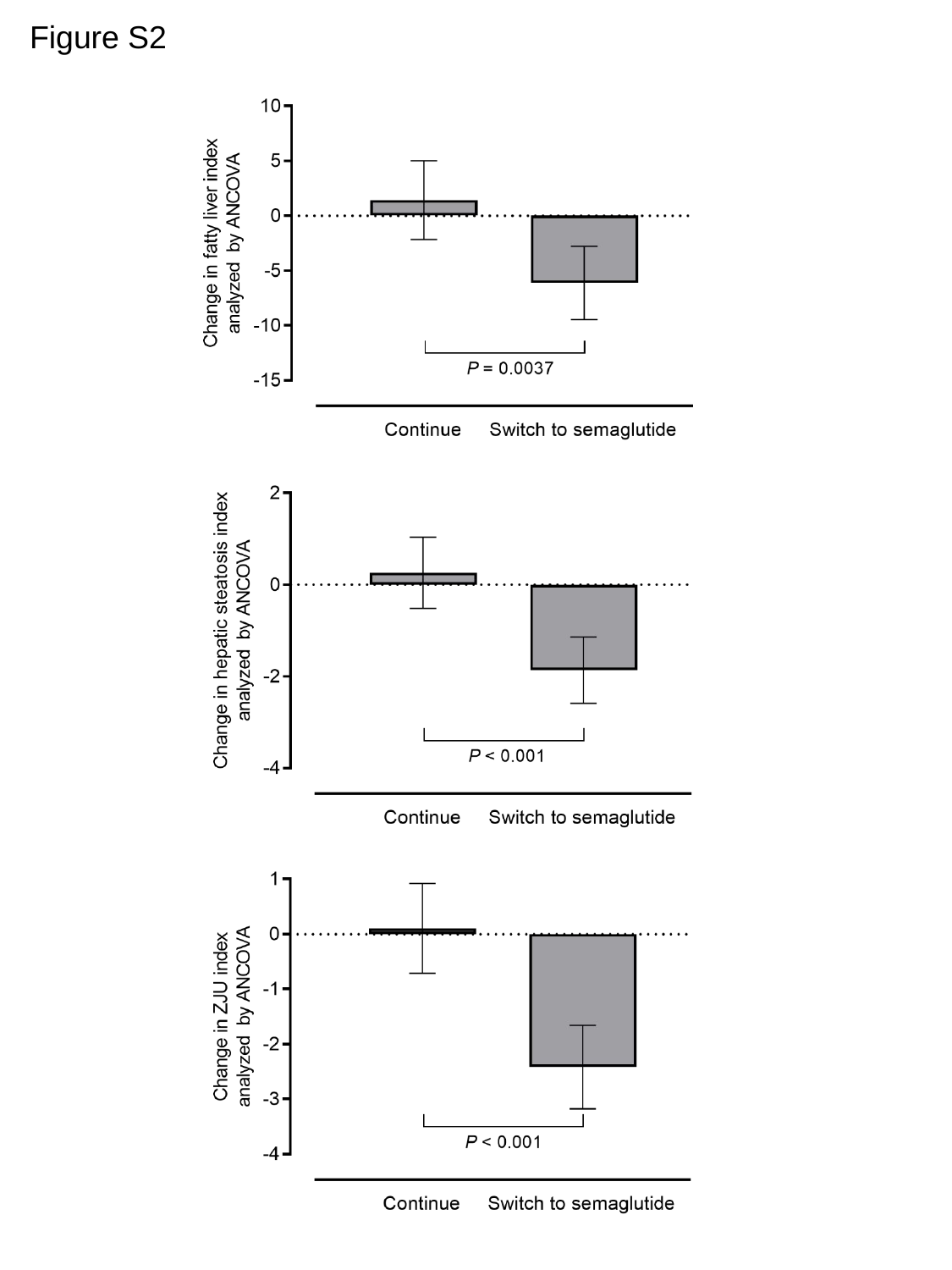

Figure S2

## Slide 3
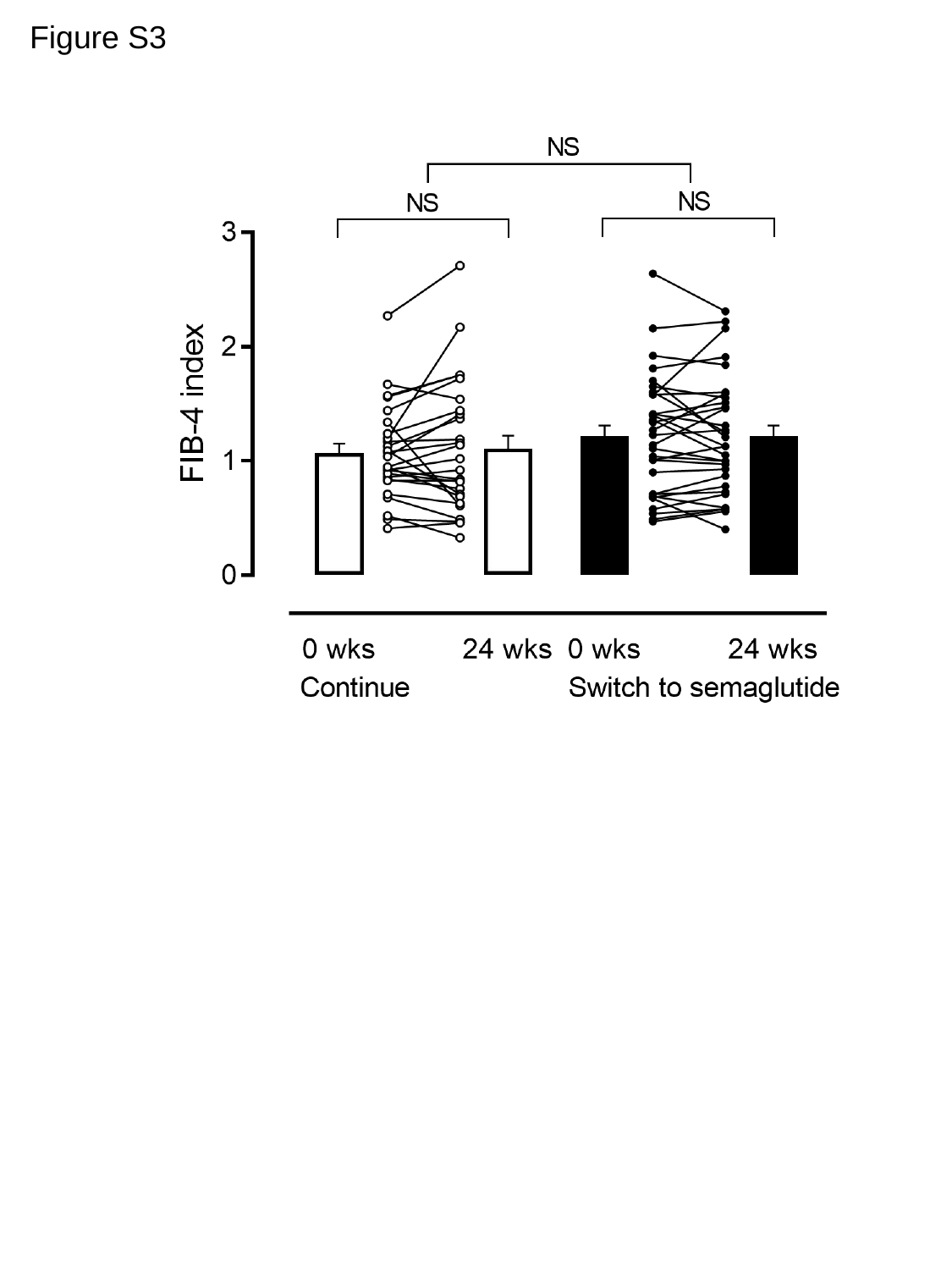

Figure S3

## Slide 4
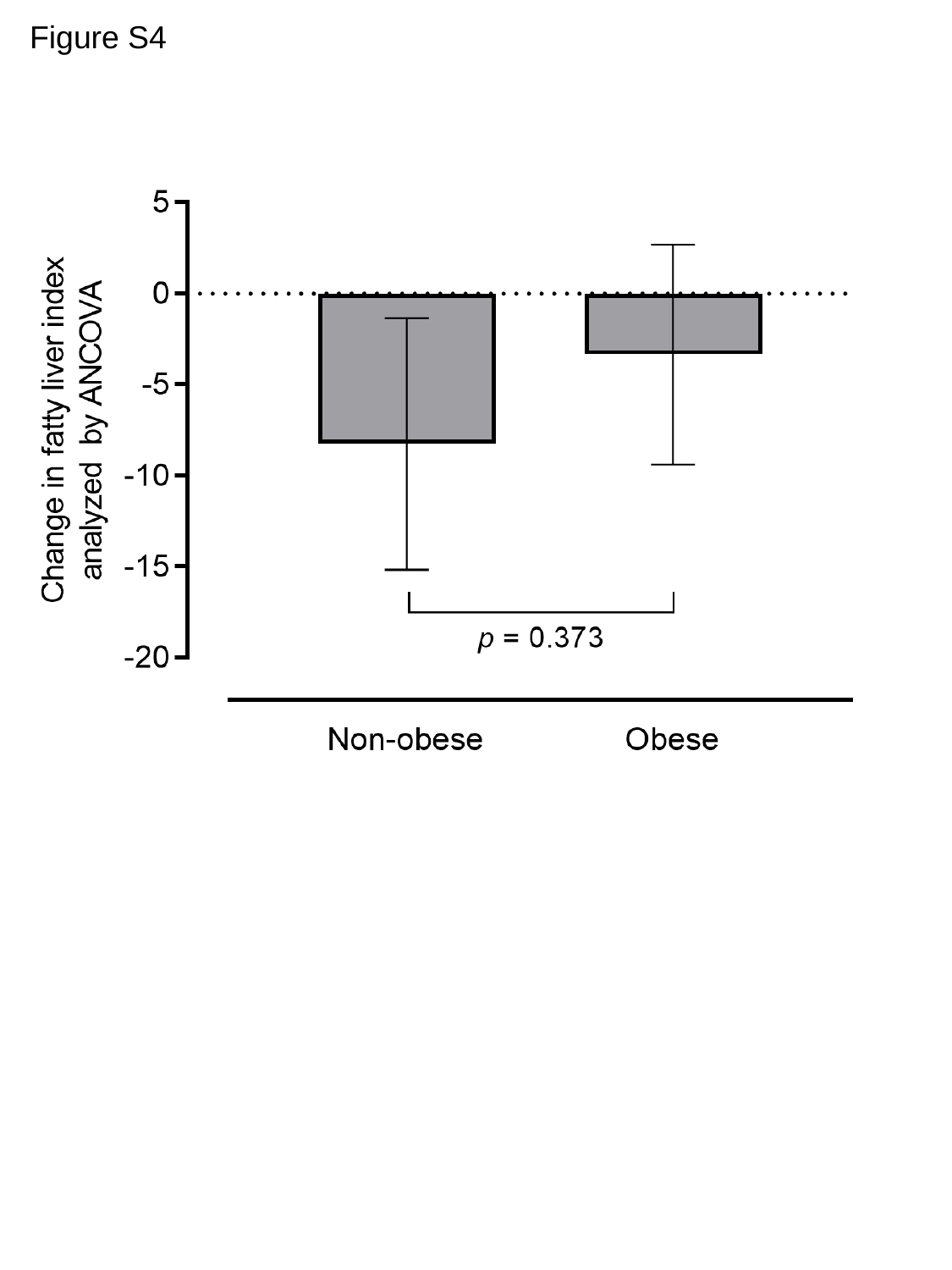

Figure S4

Supplement: Supplementary file 1 [file pharmaceutics-15-02163-s001.zip › Supple Figures_20230815.pptx]
